# Acceptability of screening for celiac disease at Youth Health Care Centers in The Netherlands

**DOI:** 10.1007/s00431-026-06809-6

**Published:** 2026-04-06

**Authors:** Willemien de Vries, Nan van Geloven, M. Luisa Mearin, Lucy Smit, Martine C. de Vries, Caroline R. Meijer-Boekel

**Affiliations:** 1 Department of Pediatric Gastroenterology, Leiden University Medical Center, Willem Alexander Children’s Hospitall, Leiden, The Netherlands; 2 Department of Medical Statistics, Leiden University Medical Center, Leiden, the Netherlands; 3 Youth Health Care Centre, Heemstede, Region of Kennemerland The Netherlands; 4 Department of Medical Ethics & Health Law, Leiden University Medical Center, Leiden, The Netherlands; 5 Department of Pediatric Gastroenterology, Leiden University Medical Centre, Willem Alexander Children’s Hospital, Albinusdreef 2, Leiden, 2333 ZA The Netherlands

**Keywords:** Celiac disease, Case finding, Mass screening, POC test

## Abstract

A novel evaluation of the acceptability of screening and mass screening for celiac disease (CD) in young children at the Preventive Youth Health Care Centres (YHCCs) in the Netherlands. Children aged 1–4 years attending the YHCC Kennemerland region were screened for CD-related symptoms. Symptomatic children were offered a point-of-care (POC) test for CD antibodies. Parents of both symptomatic and asymptomatic children received tailored questionnaires assessing opinions on case finding and mass screening and about distress and anxiety. Parents of POC-positive children received follow-up questionnaires during diagnosis. Questionnaires were completed for 1070 asymptomatic children, 1095 symptomatic participants, and 653 symptomatic non-participants. Sixty-one children tested POC-positive; 39 follow-up questionnaires were returned. Reported distress and anxiety were mild, mainly after a positive POC result. Among parents who refused testing, 32% (80/248) cited practical obstacles. Of those who accepted, 88% would participate again in the future. Overall, 87% considered mass screening a good idea, but only 68% (2125/3111) would allow participation of their asymptomatic child.

*Conclusion*: Active case finding and mass screening for CD is well accepted in the Dutch population, with limited distress for parents and children. Reducing practical barriers is essential for successful implementation.

**Supplementary Information:**

The online version contains supplementary material available at 10.1007/s00431-026-06809-6.

**Table:** 

**What is Known:** • *Celiac disease (CD) is difficult to diagnose promptly, and undetected CD is associated with short- and long-term complications*. • *Strategies for secondary prevention of CD include active case finding and mass screening, but the acceptability of these strategies is not well known*.		
**What is New:** • *Active case finding for CD via a questionnaire and, when indicated, a subsequent POC test in children aged 0–4 years, is generally well accepted by parents and health care providers*. • *Parents who refused participation mentioned the absence of significant symptoms and practical barriers as the main reasons*.		

## Introduction

Celiac disease (CD) is a common autoimmune disorder, caused by the ingestion of gluten in genetically susceptible individuals [1]. It is characterized by the production of specific autoantibodies against transglutaminase type 2 (TGA) and endomysium (EMA) and affects as many as 1–3% of the general population [2, 3].

CD may present with heterogeneous and nonspecific symptoms [4]. Despite well-implemented guidelines for CD diagnosis and increasing awareness under health care professionals, the majority of affected individuals still report a delay in diagnosis that may last for years. Those with undiagnosed and thus untreated disease are at risk for short- and long-term complications [5–9].

Since it remains difficult to recognize and diagnose CD promptly, prevention of the disease would be desirable. Large prospective interventional studies have shown that primary prevention of CD, via dietary interventions, is not possible [7, 10–12]. Secondary prevention can be achieved by early detection and treatment, via case finding/screening or mass screening. However, the potential benefits and ethical issues related to population-based mass screening for CD remain a matter of debate [7, 13].

Active case finding, often called screening, refers to liberal diagnostic testing of persons with CD-associated symptoms and has been shown to improve the diagnostic rate of CD [14, 15].

Easy-to-use, onsite, point-of-care (POC) tests for detection of the specific CD antibodies against TGA have been shown to be effective in detecting undiagnosed cases. It is attractive to use in preventive care settings, where it could be used for active case finding or mass screening [16, 17].

For successful implementation of a new method for early detection, it is necessary to know the acceptance level of the target population [18]. Few studies have investigated the acceptance of methods for early detection of CD. A Swedish study in adolescents showed that mass screening for CD is accepted by those diagnosed and their parents, although they recommended screening earlier than adolescence [19]. A study in the UK showed high customer and service provider satisfaction in CD case finding via community pharmacies; however, only 8% of participants completed the satisfaction survey [20]. The parental and health care provider acceptance of early CD detection in young children in a general population has not been studied.

In The Netherlands, more than 95% of children until the age of 4 years visit the preventive Youth Health Care Centers (YHCC’s). The aim of this study is to assess for the first time in both parents and health care professionals (1) the acceptability of active case finding for CD at the YHCC in The Netherlands and (2) the opinion about mass screening for CD [21, 22].

## Methods

### GLUTENSCREEN

This novel acceptability study of case finding was conducted as part of the GLUTENSCREEN project described before [23]. Briefly, parents/legal guardians (from now on called “parents”) of all children aged 12 months to 4 years old attending a regular visit to the YHCC in the region of Kennemerland were asked to participate. Children with one or more symptoms of a standardized questionnaire based on “Who to test for CD” from the ESPGHAN guideline [1] (Appendix 2) were invited to active case finding. Before completing the symptom questionnaire, oral informed consent was requested. Written informed consent by both parents/guardians was requested before performing the POC test (Celiac Quick Test, Biohit Oyj). For this reason, the POC test was therefore performed during a second appointment. The diagnosis of CD was considered confirmed with TGA titres > 7 × upper limit of normal (Themo Fisher, Germany; ImmunoCAP250, cutoff of normality 7 U/ml) plus positive EMA results (Aeskuslides, Germany) or by small bowel biopsies [23].

### Acceptance of active case finding and mass screening

**Fig. 1:**
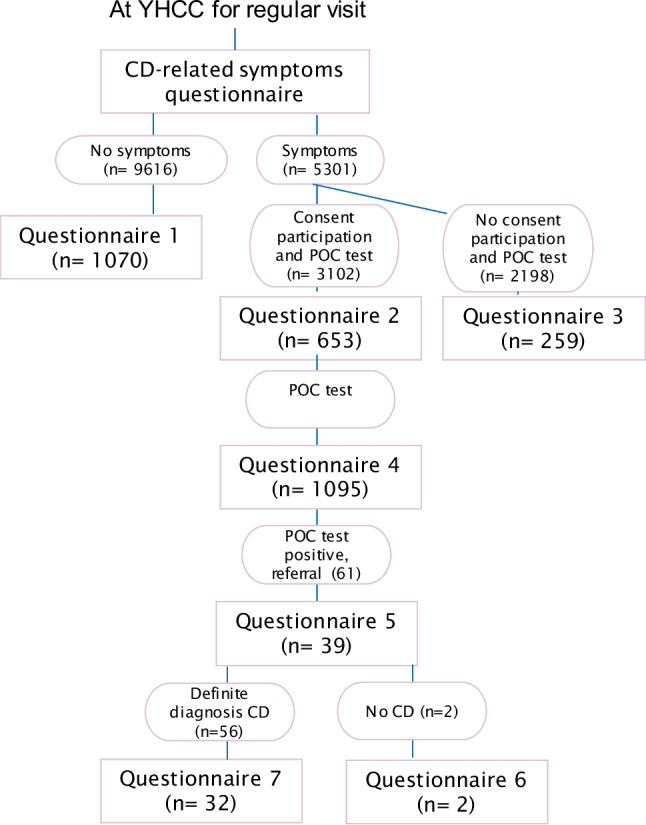
Flow chart of children participating in the GLUTENSCREEN study with the number of completed questionnaires

Parents of both symptomatic and asymptomatic children and all health care providers (*n* = 135) involved were invited to fill in questionnaires about the acceptance of active case finding for CD and about their opinion on mass screening for CD; see Fig. 1.

Parents were invited to participate from February 2019 until 4th January 2022 (inclusion was paused 19th March until 16th August 2020 due to the COVID pandemic) and health care professionals from June until August 2020. Health care professionals were sent reminders 2, 4, and 8 weeks after the initial invitation.

### Questionnaires

Since no validated questionnaire existed on the acceptance of early CD detection in young children, questionnaires were developed based on previously published ones, see appendix 1 [20, 24–26]. We distributed 7 questionnaires: for parents of asymptomatic children (Q1), for parents of symptomatic children who gave or did not give consent for CD testing (Q2 and Q3, respectively), for parents of children who underwent the POC test (Q4), for parents of children with a positive POC-test upon their first appointment with the pediatric gastroenterologist (Q5), for parents of children in whom CD was ruled out after further diagnostics (Q6), and for parents of children with a definite diagnosis of CD after further diagnostics (Q7). All questionnaires for parents were handed out on paper. In addition, one questionnaire for health care professionals was distributed via Castor Electronic Data Capture, a web-based clinical data management system.

All questionnaires were processed in Castor. Upon informed consent, patients could consent for their data to be processed coded or anonymously. Coded data could be followed throughout the consequent questionnaires.

Data collection was stopped when the number of respondents was considered representative of the study population. Data collection was stopped in October 2019 at the amount of 1070 questionnaires 1, and 653 questionnaires 2, and stopped in September 2020 at the amount of 1095 questionnaires 4.

### Ethical approval

Ethical approval was obtained from the Medical Ethics Committee of Leiden-Den Haag-Delft (METC-LDD; P17.240). Informed consent for participation in the study was obtained in conformity with Dutch legislation.

### Statistics

To assess which covariates predict participation, the presence of anxiety and depression, and acceptance of parents, univariate logistic regression was used. For all other parameters, descriptive statistics were used. A *P*-value of < 0.05 was considered statistically significant. All analysis were performed with IBM SPSS Statistics version 28 for Mac.

## Results

### GLUTENSCREEN participants

A total of 16,289 parents were approached to fill in the questionnaire on CD-related symptoms and 14,917 of them (92%) gave informed consent. Sixty-one children had a positive POC test. In two of 61 children this appeared to be a false positive POC test: their ELIA-TGA measured in serum was negative, and their human leukocyte antigen (HLA)-DQ2 and/or HLA-DQ8 was negative; thus, CD was ruled out. In 53/61 children, the diagnosis of CD was established based on serology alone [23, 27]. In 6/61 children, duodenal biopsies were required. In three of them, the diagnosis of potential CD was made, and in three of them, CD could be established [23].

### Numbers and characteristics of study participants

#### Parents

The numbers of the included acceptance questionnaires are depicted in Fig. 1. The demographic characteristics of the parents are shown in Table 1. The participating parents are representative for the region where the study took place in terms of gender, age distribution, educational level, and socioeconomic status [23]. On average, they have a higher educational level and socioeconomic status compared to the general Dutch population [23].

**Table 1:** Demographic characteristics of the parents who participated in this study

	Questionnaire 1 *N* = 1070	Questionnaire 2 *N* = 653	Questionnaire 3 *N* = 259	Questionnaire 4 *N* = 1095	Questionnaire 5 *N* = 39	Questionnaire 6 *N* = 2	Questionnaire 7 *N* = 32
Gender child							
Boy	51% (498/969)	55% (242/443)	59% (99/168)	52% (555/1070)	26% (10/38)	50% (1/2)	20% (6/30)
Girl	49% (471/969)	45% (201/443)	41% (69/168)	48% (515/1070)	74% (28/38)	50% (1/2)	80% (24/30)
Age children in months, mean (SD)	28.3 (10.9)	26.1 (11)	24.3 (10.4)	25.5 (10.9)	30.4 (10.7)	48 (NA)	31.1 (10.7)
Country of birth parent 1							
Netherlands	91% (722/791)	92% (397/432)	93% (142/152)	90% (933/1033)	97% (35/36)	100% (2/2)	90% (27/30)
Other	9% (69/791)	8% (35/432)	7% (10/152)	10% (100/1033)	3% (1/36)	0% (0/2)	10% (3/30)
Country of birth parent 2							
Netherlands	88% (686/782)	87% (366/421)	92% (131/143)	88% (876/997)	92% (33/36)	0% (0/2)	93% (28/30)
Other	12% (96/782)	13% (55/421)	8% (12/143)	12% (121/997)	8% (3/36)	100% (2/2)	7% (2/30)
Educational level parent 1*							
No education	0% (1/1060)	0% (0/449)	0% (0/173)	0% (0/1083)	0% (0/17)	NA**	0% (0/14)
Low	9% (98/1060)	7% (30/449%)	5.2% (9/173)	6% (65/1083)	0% (0/17)	NA	0% (0/14)
Intermediate	26% (271/1060)	23% (103/449)	21.4% (37/173)	25% (267/1083)	29% (5/17)	NA	0% (0/14)
High	65% (689/1060)	70% (316/449)	73.4% (127/173)	69% (746/1083)	71% (12/17)	NA	100% (14/14)
Educational level parent 2*							
No education	0% (3/1023)	0% (2/431)	0% (0/165)	1% (5/1045)	0% (0/17)	NA	0% (0/14)
Low	9% (91/1023)	8% (36/431)	12% (19/165)	8% (82/1045)	6% (1/17)	NA	7% (1/14)
Intermediate	26% (263/1023)	24% (104/431)	25% (42/165)	25% (260/1045)	12% (2/17)	NA	0% (0/14)
High	65% (663/1023)	67% (287/431)	63% (104/165)	67% (697/1045)	82% (14/17)	NA	93% (13/14)

*Q1*, parents of asymptomatic children; *Q2*, parents of symptomatic children whose parents gave consent for CD testing; *Q3*, parents of symptomatic children whose parents did not give consent for CD testing; *Q4*, parents of children who underwent the POC test; *Q5*, parents of children with a positive POC-test upon their first appointment with the pediatric gastroenterologist; *Q6*, parents of children in whom CD was ruled out after further diagnostics; *Q7*, parents of children with a definitive diagnosis of CD after further diagnostics

^*^Educational levels defined as: low, vocational education; intermediate, secondary education; high, higher professional education or university

^**^NA, not answered

Not all questionnaire items were answered by the participants; see appendix 3 for the number of answers for each. All stated percentages are based on these numbers.

#### Health care professionals

The response rate of the health care professionals was 58% (78/135). Of the responding health care professionals, 10% were vaccinators (8/78), 26% youth doctors (20/78), 13% assistants (10/78), 47% nurses (37/78), 4% managers (3/78), and 8% (6/78) planners. Because some health care professionals had multiple roles, the sum adds up above 100%.

### Acceptance of active case finding by participants

#### Parents

Among parents of asymptomatic children (who therefore did not get the POC test), 90% (954/1055) would have allowed their child to participate in the case finding if symptomatic, 6% (66/1055) would not, and 3% (35/1055) had no opinion. Among parents whose children underwent the POC test, 88% (953/1083) would participate in an early detection test for CD again in the future, 4% (45/1083) would not, and 8% (85/1083) had no opinion. Distress or anxiety did not affect these results. Among parents of children seen in the LUMC for diagnostics according to standard of care, 92% (36/39) would allow future participation in an early detection test for CD, 3% (1/39) would not, and 5% (2/39) had no opinion.

#### Health care professionals

When asked if early detection of CD adds value to the care provided by the YHCC for children aged 1–4 years, 99% (77/78) of healthcare professionals agreed or strongly agreed, with no significant differences based on their role. Regarding the feasibility of implementing the case-finding screening tasks during a regular consultation, 50% agreed (39/78).

### Predictors of participation in case finding

Worries about their child’s health and suspicion that their child might have CD were found to be significant predictors for participation (*P* = 0.012 and *P* < 0.001 respectively; OR for decline participation = 0.188, 95% CI = 0.089–0.395; OR = 0.074, 95% CI = 0.023–0.240). The age and gender of the child, educational level and country of birth of the parents, and satisfaction with information received were not significant predictors for participation.

Reasons for declining the POC test were in 75% (181/240) that symptoms were not serious enough from the parent’s point of view, 8% (20/240) found the test too invasive, 6% (15/240) were previously tested for CD, 5% (13/240) because of logistic reasons, and 5% (11/240) gave various other reasons. Of all parents who did not consent, 32% (80/248) indicated they would have consented if the POC test was done immediately during their initial visit to the YHCC.

#### Distress and anxiety in active case finding

Few parents of asymptomatic children (2%, 22/1061) reported to be worried about their child’s health in contrast to 20% (130/640) of the parents with symptomatic children (*P* < 0.001). The parents of a symptomatic child were more likely to participate when the parents were worried about the child’s health, 4% (11/258) of the parents who declined participation were worried, and 17% (179/1083) of the parents whose child underwent the POC test were worried (*P* < 0.001).

The majority of parents reported to have confidence in the POC test (92%, 998/1088), 0.6% (7/1088) did not, and 8% (83/1088) stated to not have an opinion. Most parents (88%, 954/1086) reported not to be worried about the result of the POC test, 5% (56/1086) reported to be worried, and 7% (76/1086) did not have an opinion. Of the parents who reported to be worried about the health of their child, 70% (160/232) reported not to be worried about the result of the POC test, and 14% (32/232) reported to be worried, 17% had no opinion (40/232). On a scale from 1 to 10, the mean satisfaction about the information received while waiting for the result of the POC test was 8.4 ± 1. 1 SD.

A positive POC test caused some degree of distress in many of the parents (Table 2), and significantly more than among the parents whose child had a negative test. However, a negative result did not resolve the worries in all the parents; 28% reported to be “not reassured,” and 43% to be “not relieved.” Age and gender of child, country of birth, and educational level of the parents and satisfaction with the received information were not significant predictors for distress or anxiety.

**Table 2:** Distress and anxiety in parents whose children participated in case finding for celiac disease, according to POC test result for TGA

POC result	Shocked	Concerned	Anxious	Unhappy	Not reassured	Not relieved
Positive	72.2% (26/36)	73.5% (25/34)	45.7% (16/35)	56.2% (18/32)	71.8% (23/32)	84.8% (28/33)
Negative	7.1% (51/718)	11.1% (78/704)	7.6% (53/694)	9.5% (66/693)	28% (241/850)	43.1% (356/826)
*Chi-square test*	*P* = 0.00	*P* = 0.00	*P* = 0.00	*P* = 0.00	*P* = 0.00	*P* = 0.00

Percentages add up to > 100% due to the possibility of expressing multiple feelings

Feelings of parents whose children were referred to LUMC for further diagnostics because of a positive POC test are listed in Table 3. Most parents reported no tense feelings and/or worries and that they usually felt relaxed and cheerful.

**Table 3:** Anxiety and depression feelings by parents whose children had a positive POC test result for celiac disease and were referred for diagnostics according to the standard of care

		Before definitive diagnosis (*N* = 39)	After CD confirmation (*N* = 32)	After CD ruled out (*N* = 2)
I felt tense	Usually	0% (0/39)	0% (0/32)	0% (0/0)
	Regularly	15.4% (6/39)	9.4% (3/32)	0% (0/0)
	Rarely	56.4% (22/39)	46.9% (15/32)	0% (0/0)
	Not at all	28.2% (11/39)	43.8% (14/32)	100% (2/2)
I felt relaxed	Surely	38.5% (15/39)	43.8% (14/32)	100% (2/2)
	Usually	46.2% (18/39)	46.9% (15/32)	0% (0/0)
	Seldom	12.8% (5/39)	9.4% (3/32)	0% (0/0)
	Not at all	2.6% (1/39)	0% (0/0)	0% (0/0)
I was worried	Very often	2.6% (1/39)	3.1% (1/32)	0% (0/0)
	Often	10.3% (4/39)	0% (0/0)	0% (0/0)
	Rarely	56.4% (22/39)	65.6% (21/32)	0% (0/0)
	Occasionally	30.8% (12/39)	31.3% (10/32)	100% (2/2)
I felt cheerful	Not at all	0% (0/39)	0% (0/0)	0% (0/0)
	Seldom	7.7% (3/39)	0% (0/0)	0% (0/0)
	Sometimes	10.3% (4/39)	6.3% (2/32)	0% (0/0)
	Usually	82.1% (32/39)	93.8% (30/32)	100% (2/2)

### Acceptance of mass screening

When asked about mass screening in general, 79% (2448/3112) considered it a good idea. However, when asking about participation if their child was asymptomatic, only 68% (2125/3111) expressed willingness to participate. Table 4 shows differences in acceptance between the different parents’ groups.

**Table 4:** The acceptance of mass screening on CD in the various studied groups

		Q1 (*n* = 1060)	Q2 (*n* = 647)	Q3 (*n* = 253)	Q4 (*n* = 1085)	Q5 (*n* = 39)	Q6 (*n* = 2)	Q7 (*n* = 32)
Mass screening is a good idea, %	Yes	74.5% (790/1060)	83.5% (540/647)	65.6% (166/253)	82.4% (889/1079)	84.6% (33/39)	50% (1/2)	90.6% (29/32)
	No	9.4% (100/1060)	5.3% (34/647)	16.2% (41/253)	5.6% (60/1079)	2.6% (1/39)	50% (1/2)	6.3% (2/32)
	No opinion	16.0% (170/1060)	11.3% (73/647)	18.2% (46/253)	12.0% (130/1079)	12.8% (5/39)	0% (0/2)	3.1% (1/32)
Test my child without symptoms, %	Yes	66.4% (702/1057)	71.4% (461/646)	33.6% (84/250)	75.5% (819/1085)	84.6% (33/39)	50% (1/2)	78.1% (25/32)
	No	22.7% (240/1057)	20.0% (129/646)	51.2% (128/250)	15.9% (173/1085)	12.8% (5/39)	50% (1/2)	15.6% (5/32)
	No opinion	10.9% (115/1057)	8.7% (56/646)	15.2% (38/250)	8.6% (93/1085)	2.6% (1/39)	0% (0/2)	6.3% (2/32)

*Q1*, parents of asymptomatic children; *Q2*, parents of symptomatic children whose parents gave consent for CD testing. *Q3*, parents of symptomatic children whose parents did not give consent for CD testing; *Q4*, parents of children who underwent the POC test; *Q5*, parents of children with a positive POC test upon their first appointment with the pediatric gastroenterologist; *Q6*, parents of children in whom CD was ruled out after further diagnostics; *Q7*, parents of children with a definite diagnosis of CD after further diagnostics

Appendix 4 presents the answers of parents to the open question about their opinions on mass screening, including those willing and unwilling to test their asymptomatic child. In both groups, answers concerning individual certainty and diagnostics were the most prevailing. Also, a notable proportion of parents (10–25%) gave answers that could not be categorized in one of the main categories, and 20–45% had no opinion or left the question blank, particularly among parents who would not test. Child’s age and gender, parental educational level, country of birth, presence of symptoms, and satisfaction with the information provided about the study were not significant predictors for acceptance of mass screening.

## Discussion

To our knowledge, this is the first study on parent acceptability regarding active case finding and mass screening for CD in young children. The results show that, among the Dutch population, active case finding for CD is well accepted, with parents showing a positive attitude towards mass screening as well.

The POC test for the detection of TGA antibodies has proven to be a reliable, easy-to-use, and non-invasive test [16, 27], making it suitable for active case finding or mass screening in countries with appropriate infrastructure for child health care. In the current study, a high percentage of parents (92%) expressed confidence in the POC test.

The participation rate in this active case finding study was 59%. Practical barriers, such as the requirement for written informed consent from both parents for research purposes and the necessity for a second appointment for the POC test, likely contributed to a drop in participation. Even though only 5% of the parents reported this as the main reason to refuse participation, one-third of all refusing parents indicated that they would have participated if the POC test had been performed immediately. At the YHCC of the Kennemerland region, where the case finding strategy for CD is now standard care, 21 confirmed cases of CD were found in 811 tested children (2.6%, personal communication). This could suggest a higher participation rate of symptomatic children than during the GLUTENSCREEN study, where 1.7% of tested children had a confirmed CD diagnosis. However, the higher diagnostic rate might also be influenced by growing awareness of the topic in the region, the increase of autoimmune disorders, perhaps the impact of COVID pandemia, or randomness [28].

Of the 6 children who required duodenal biopsies, 3 of them were found to have potential CD, which is a higher percentage than generally reported in the literature. This might be explained by early disease interception through screening and young age at diagnosis.

In a screening study among first-degree relatives of CD patients, the participation rate was higher (70%) [29]. The difference might be explained by the fact that these participants were already familiar with CD, its genetic component, and consequences, whereas the population of the current study is generally healthy and might not have had specific knowledge regarding CD.

Parents who refused the POC test often cited the absence of significant symptoms in their child, even when their child scored positive on the CD-related symptoms checklist. It appears that many parents are not particularly alarmed by symptoms such as abdominal pain or diarrhea.

Interestingly, a significant number of parents felt “not reassured” and “not relieved” after receiving a negative POC test result. This may be explained because the act of completing the symptom checklist itself might raise distress and anxiety, prompting parents to think about their child’s health.

In this study, 3.3% of the POC tests gave false positive results. A false positive result can lead to heightened distress and unnecessary health care consumption [30, 31]. Both effective communication about the potential outcomes and the possibility to perform subsequent testing soon after the first result might decrease the levels of distress [30, 32].

It is worth noting that “distended abdomen” is the only symptom clearly discriminative for CD [23], suggesting that a symptom checklist may not enhance diagnostic accuracy but could contribute to increased distress. This raises the possibility that a mass screening strategy, where symptoms are not asked, might be less-anxiety-inducing for the target population. Furthermore, when HLA typing would be incorporated into mass screening, around 60% of the population will have a HLA-type other than DQ2 and/or DQ8 and can be told that their child’s risk to develop CD is negligible.

Studies have shown that both active case finding and mass screening for CD are cost-effective compared to standard care, with mass screening being the most cost-effective [33]. Implementing either active case finding or mass screening for CD can improve overall health by preventing the potential long-term consequences associated with delayed diagnosis [6]. Most newly diagnosed celiac cases are identified before pubertal age [34]. Further studies should clarify which age groups are to be addressed in active case finding or mass screening. Almost all health care professionals in our study (strongly) agreed that active case finding for CD added value to care provided at the YHCCs. It is important that they should be given adequate support in terms of time and finance to conduct the POC test.

Strict compliance with a lifelong gluten-free diet (GFD) can present significant challenges, especially for children who do not experience symptoms and adolescents. In our study, the children detected with CD were very young, and it is assumed that the younger the initiation of a GFD, the better acceptance [35].

Regarding mass screening for CD, we found that the willingness to participate is lower when the child is asymptomatic, and even lower in the group who refused participation in active case finding. Participation rates are lower than those, for example the heel prick, presumably because in the case of CD parents feel more capable of assessing their child’s symptoms and do not consider screening for CD as a single test opportunity.

In Italy, a law introducing a national mass screening program for CD and diabetes type 1 by testing for disease-specific antibodies was approved in 2024 [36]. In the state of Colorado (USA), there is an active mass screening program on CD and T1D [37, 38].

As a result of GLUTENSCREEN and the current study, plans have been made to expand this strategy to all YHCC across The Netherlands. Debate regarding these plans with governmental organisations is ongoing.

It will be of great interest to learn how many parents make use of those screening initiatives and what factors could improve their participation. More on the long term, the actual and perceived health benefits should be studied.

A potential limitation of this study is the possibility of bias in the survey responses, as parents who favor screening may have been inclined to complete the questionnaires [39].

Strength is the inclusion of a large study group from the general population, which enhances the generalizability of the findings to the Dutch population. Additionally, by including parents of asymptomatic children and those who declined participation, the study provides valuable insights into attitudes across the target population.

## Conclusion

Our novel data show that active case finding for CD in the Dutch YHCC setting is highly accepted by parents of young children. Although some parents, especially those with a positive POC test, report feelings of distress and anxiety, they are willing to participate again in the future. In general, parents have a positive attitude towards mass screening for CD. Health care providers are strong advocates of CD testing in young children. To achieve high participation rates, clear information about the disease and the testing process, and testing immediately after consultation, are essential.

## Supplementary Information

Below is the link to the electronic supplementary material.

ESM 1 (PDF 622 KB)

ESM 2 (DOCX 25.0 KB)

ESM 3 (DOCX 16.7 KB)

ESM 4 (DOCX 16.5 KB)

## Data Availability

No datasets were generated or analysed during the current study.

## Abbreviations

CD: Celiac disease
GFD: Gluten-free diet
HLA: Human leukocyte antigen
POC: Point of care
TGA: Tissue transglutaminase
YHCC: Youth Health Care Center
