# Supplementary material for: Acceptability of screening for celiac disease at Youth Health Care Centers in The Netherlands
Source: Eur J Pediatr. 2026 Apr 6;185(5):237. doi: 10.1007/s00431-026-06809-6 (PMC13053595; doi:10.1007/s00431-026-06809-6)
Supplement: Supplementary file 1 — (PDF 622 KB) [file 431_2026_6809_MOESM1_ESM.pdf]

Appendix one

All Questionnaires below are translations from the original Dutch questionnaires for publication purposes. All parents/guardians and health care professionals received the Dutch version of the questionnaires.

Questionnaire 1 – No Complaints (Case-Finding Group)

Dear Parent(s)/Guardian(s),

We are pleased to hear that your child has NO symptoms suggestive of celiac disease!
Celiac disease is a chronic intolerance to gluten. However, this does not completely rule out celiac disease, as it can also occur without symptoms.
Currently, it is not possible in the Netherlands to test all children (with or without symptoms) who visit the Youth Health Care Centre for celiac disease. Only children with symptoms may be tested.
We would like to know your opinion on this. Please answer a few short questions. The questionnaire takes a maximum of 5 minutes and can be completed anonymously.

For questions, contact:

- JGZ Kennemerland: (phone number present in original questionnaire)
- LUMC: (e-mail address and phone number present in original questionnaire)

Thank you very much for your time and cooperation!

Child's Age: (years/months) **Sex:** ☐ boy ☐ girl

Country of Birth: Parent 1 ______ Parent 2 ______

What is the highest level of education completed by the parent(s) with a diploma?

**Level of Education**	**Parent 1**	**Parent 2**
No education (did not finish primary)	☐	☐
Primary education	☐	☐
Lower/pre-vocational secondary (e.g., LTS, LEAO, VMBO-p)	☐	☐
General secondary education (e.g., MAVO, MBO-short)	☐	☐
Vocational education (e.g., MBO-long, MTS)	☐	☐
Higher general/pre-university (e.g., HAVO, VWO)	☐	☐
Higher professional education (HBO)	☐	☐
University education	☐	☐
Other, namely: ______		

We would like your opinion on the research study "early detection of celiac disease in Youth Health Care Centres," called GLUTENSCREEN.

Please mark the box that best fits your answer:

Question	Yes	No	No Opinion
1. Are you concerned about your child's health?	☐	☐	☐
2. Do you suspect your child may have celiac disease?	☐	☐	☐
3. If your child HAD symptoms, would you consent to a finger prick test?	☐	☐	☐
4. Do you think a mass screening for celiac disease (testing all children) is a good idea?	☐	☐	☐
5. If it had been possible to test children without symptoms, would you have done so (knowing 4 out of 10 celiac cases have no symptoms)?	☐	☐	☐

Can you explain your answer to question 5 below?
[Open field]

Do you have any questions or comments about the research or this questionnaire?
[Open field]

Thank you for your cooperation!

Questionnaire 2 – With Complaints (Case-Finding Group)

Dear Parent(s)/Guardian(s),

You just had an appointment at the Youth Health Care Centre with a nurse or youth doctor. During this visit, you answered questions about your child's health. It appeared your child has **one or more** complaints that may be related to celiac disease, a gluten intolerance.

In the Netherlands, only children with complaints can currently be tested for this disease at the Youth Health Care Centre. We would like to know your opinion. Therefore, we ask you to answer a few questions about early detection of celiac disease. This will take no more than 5 minutes.

☐ Yes, I will participate! I want to share my opinion and allow the use of my personal data (age, gender, country of birth, education) and my child's complaints questionnaire. These will be processed using coded data.
☐ Yes, I will participate, but I DO NOT want my or my child's data to be used. My answers will be processed anonymously.
☐ No, I do not want to participate.

Child's Age: (years/months) **Sex:** ☐ boy ☐ girl

Country of Birth: Parent 1 ______ Parent 2 ______

What is the highest level of education completed by the parent(s) with a diploma?

**Level of Education**	**Parent 1**	**Parent 2**
No education (did not finish primary)	☐	☐
Primary education	☐	☐
Lower/pre-vocational secondary (e.g., LTS, LEAO, VMBO-p)	☐	☐
General secondary education (e.g., MAVO, MBO-short)	☐	☐
Vocational education (e.g., MBO-long, MTS)	☐	☐
Higher general/pre-university (e.g., HAVO, VWO)	☐	☐
Higher professional education (HBO)	☐	☐
University education	☐	☐
Other, namely: ______		

We would like to know your opinion about the study 'early detection of celiac disease at Youth Health Care Centers,' GLUTENSCREEN. Please check the box that best matches your answer for the following questions. At the end of the questionnaire, there is space for additional questions/comments.

For questions, contact:

- JGZ Kennemerland: (phone number present in original questionnaire)
- LUMC: (e-mail address and phone number present in original questionnaire)

Thank you very much for your time and cooperation

Questions:

1. Are you concerned about your child's health?
   ☐ Yes ☐ No ☐ No opinion
2. Do you suspect your child has celiac disease?
   ☐ Yes ☐ No ☐ No opinion
3. Have you contacted healthcare providers due to the complaints?
   ☐ Yes → Go to Q4
   ☐ No → Go to Q7
4. If yes, who?
   ☐ Youth doctor/nurse
   ☐ General practitioner
   ☐ Pediatrician
   ☐ Pediatric gastroenterologist
   ☐ Dietitian
   ☐ Other: ______
   How often? [fill in]
5. Were any tests conducted?
   ☐ Yes → Go to Q6
   ☐ No → Go to Q7
6. What types of tests?
   ☐ Blood
   ☐ Urine
   ☐ Stool
   ☐ Ultrasound
   ☐ Other: ______
   How often? [fill in]
7. Were you sufficiently informed about the celiac screening in our study?
   ☐ Yes → Go to Q8
   ☐ No
8. Information evaluation:
   ☐ Yes, clear and complete
   ☐ No:
   - I wanted more info about why this research is done
   - I wanted more info on what the test entails

- I wanted the info earlier
- I received too much info
- Other: ______

9. Do you think mass screening for celiac disease is a good idea (test all children)?
   ☐ Yes ☐ No ☐ No opinion
10. Would you have tested your child if there were no complaints, knowing 4 in 10 celiac patients have no symptoms?
    ☐ Yes ☐ No ☐ No opinion

Please explain your answer to Q10: [open field]

Any other comments or questions? [open field]

Questionnaire 3 – With Complaints, No Consent for Finger Prick (POC Test)

Dear Parent(s)/Guardian(s),

You just had a consultation at the Youth Health Care Centre with the nurse or youth doctor. You answered questions about the health of your child. Your child has **one or more** symptoms that could indicate celiac disease. Celiac disease is a gluten intolerance.

In the Netherlands, only children with complaints can currently be tested for this disease at the Youth Health Care Centre. At the clinic, children with symptoms are offered a finger prick test. Participation is optional. You have chosen not to have the test done.

We would like to understand your choice. Your opinion is very important for our research. Therefore, we ask you to answer a few questions about early detection of celiac disease. This will take no more than 5 minutes.

☐ Yes, I will participate! I want to share my opinion and allow the use of my personal data (age, gender, country of birth, education) and my child's complaints questionnaire. These will be processed using coded data.
☐ Yes, I will participate, but I DO NOT want my or my child's data to be used. My answers will be processed anonymously.
☐ No, I do not want to participate.

Date & Signature
Date: ______ Signature: ______

Child's Age: (years/months) **Sex:** ☐ boy ☐ girl

Country of Birth: Parent 1 ______ Parent 2 ______

We would like to know your opinion about the study 'early detection of celiac disease at Youth Health Care Centers,' GLUTENSCREEN. Please check the box that best matches your answer for the following questions. At the end of the questionnaire, there is space for additional questions/comments.

For questions, contact:

- JGZ Kennemerland: (phone number present in original questionnaire)
- LUMC: (e-mail address and phone number present in original questionnaire)

Thank you very much for your time and cooperation

Questions:

1. Are you concerned about your child's health?
   ☐ Yes ☐ No ☐ No opinion

2. Do you suspect your child has celiac disease?
   ☐ Yes ☐ No ☐ No opinion
3. Were you sufficiently informed about the screening?
   ☐ Yes → Go to Q5
   ☐ No → Go to Q4
4. What information was lacking?
   ☐ Why the study is done
   ☐ What the testing involves
   ☐ I wanted the info earlier
   ☐ I received too much info
   ☐ Other: ______
5. Why did you decline the finger prick test? [open field]
6. If the test could have been done immediately, would you have agreed?
   ☐ Yes ☐ No ☐ No opinion
7. Do you think mass screening is a good idea?
   ☐ Yes ☐ No ☐ No opinion
8. Would you have tested your child without complaints, knowing 4 in 10 have no symptoms?
   ☐ Yes ☐ No ☐ No opinion

Explain your answer to Q8: [open field]

What is the highest level of education completed by the parent(s) with a diploma?

**Level of Education**	**Parent 1**	**Parent 2**
No education (did not finish primary)	☐	☐
Primary education	☐	☐
Lower/pre-vocational secondary (e.g., LTS, LEAO, VMBO-p)	☐	☐
General secondary education (e.g., MAVO, MBO-short)	☐	☐
Vocational education (e.g., MBO-long, MTS)	☐	☐
Higher general/pre-university (e.g., HAVO, VWO)	☐	☐
Higher professional education (HBO)	☐	☐
University education	☐	☐
Other, namely: ______		

Questions or comments: [open field]

Questionnaire 4a+b – With Complaints, Consent Given, Before and After Finger Prick Result

Dear Parent(s)/Guardian(s),

You just had a consultation at the Youth Health Care Centre with the nurse or youth doctor. You answered questions about the health of your child. The growth of your child was reviewed. Your child has **one or more** symptoms that could indicate celiac disease. Celiac disease is a gluten intolerance.

You have given permission for a finger prick (rapid test). This allows for a quick assessment of whether your child has celiac disease. The test has just been performed. You are now waiting for the result.

At present, it is only possible in the Netherlands to detect this disease in children with symptoms at the Youth Health Care Centre. We would like to know your opinion on this. Therefore, we would like to ask you a few more questions. These questions are about your views on early detection of celiac disease. Completing the questionnaire will take no more than 5 minutes.

We would like to know your opinion about the study 'early detection of celiac disease at Youth Health Care Centers,' GLUTENSCREEN. Please check the box that best matches your answer for the following questions. At the end of the questionnaire, there is space for additional questions/comments.

For questions, contact:

- JGZ Kennemerland: (phone number present in original questionnaire)
- LUMC: (e-mail address and phone number present in original questionnaire)

Thank you very much for your time and cooperation

Child's Age: (years/months) **Sex:** ☐ boy ☐ girl

Country of Birth: Parent 1 ______ Parent 2 ______

Before Result:

1. Are you concerned about your child's health? ☐ Yes ☐ No ☐ No opinion
2. Do you suspect your child has celiac disease? ☐ Yes ☐ No ☐ No opinion
3. Were you well informed about the study?
   ☐ Yes → Go to Q5
   ☐ No → Go to Q4
4. What more information would you have liked?
   ☐ Why this study is being done
   ☐ What the test involves
   ☐ Earlier information

☐ I received too much information

☐ Other: ______

5. Do you trust the reliability of the rapid test?
   ☐ Yes ☐ No ☐ No opinion
6. While waiting for the result, are you worried?
   ☐ Yes ☐ No ☐ No opinion

After Result:

7. How would you rate the information received about the result?: (Scale 1-10)
   Very bad 1 --- 1½ --- 2 --- 2½ --- 3 --- 3½ --- 4 --- 4½ --- 5 --- 5½ --- 6 --- 6½ --- 7 --- 7½ --- 8 --- 8½ --- 9 --- 9½ ---10 Excellent
8. What was the test result?
   ☐ Positive – Signs of celiac disease found
   ☐ Negative – No signs of celiac disease found
9. How did you feel after the result?
   Not shocked ☐ ☐ ☐ ☐ ☐ Very shocked
   Not worried ☐ ☐ ☐ ☐ ☐ Very worried
   Not anxious ☐ ☐ ☐ ☐ ☐ Very anxious
   Not unhappy ☐ ☐ ☐ ☐ ☐ Very unhappy
   Reassured ☐ ☐ ☐ ☐ ☐ Not reassured
   Relieved ☐ ☐ ☐ ☐ ☐ Not relieved
10. Would you participate again in early celiac screening?
    ☐ Yes ☐ No ☐ No opinion
11. Did you take time off work for the appointment?
    ☐ Yes → Go to Q12 ☐ No
12. If yes, how much time? ___ hours ___ minutes
13. Is mass screening for celiac disease a good idea?
    ☐ Yes ☐ No ☐ No opinion
14. Would you test your child without symptoms, knowing 40% have no complaints?
    ☐ Yes ☐ No ☐ No opinion
    Explain your answer to Q14: [open field]

What is the highest level of education completed by the parent(s) with a diploma?

Level of Education	Parent 1	Parent 2
No education (did not finish primary)	☐	☐
Primary education	☐	☐
Lower/pre-vocational secondary (e.g., LTS, LEAO, VMBO-p)	☐	☐
General secondary education (e.g., MAVO, MBO-short)	☐	☐
Vocational education (e.g., MBO-long, MTS)	☐	☐
Higher general/pre-university (e.g., HAVO, VWO)	☐	☐
Higher professional education (HBO)	☐	☐

**Level of Education**	**Parent 1**	**Parent 2**
University education	☐	☐
Other, namely: ______		

Comments or questions: [open field]

Questionnaire 5 – With Complaints, Positive POC Test, Before Hospital Results

Dear Parent(s)/Guardian(s),

Recently, your child had a finger prick test to measure antibodies against gluten. This was done because your child has one or more symptoms that may be related to celiac disease, which is a sensitivity to gluten.

The test indicated that your child probably has celiac disease. To confirm this, further testing is required. This additional testing will take place at the hospital: the Leiden University Medical Center (LUMC).

Currently, in the Netherlands, it is only possible to detect this disease in children with symptoms at the Youth Health Care Center. We would like to know your opinion about this. Therefore, we would like to ask you a few more questions. These questions are about your opinion on early detection of celiac disease. Completing the questionnaire takes no more than 5 minutes.

For questions, contact:

- JGZ Kennemerland: (phone number present in original questionnaire)
- LUMC: (e-mail address and phone number present in original questionnaire)

Thank you very much for your time and cooperation

Demographic Information:
Name of child: ______ Age: (years/months): ______ Sex: ☐ boy ☐ girl
Country of Birth: Parent 1 ______ Parent 2 ______

We would like to know your opinion about the study 'early detection of celiac disease at Youth Health Care Centers,' GLUTENSCREEN. Please check the box that best matches your answer for the following questions. At the end of the questionnaire, there is space for additional questions/comments.

Survey Questions

1. After the rapid test result, did you seek more information about celiac disease?
   ☐ Yes → Go to Q2
   ☐ No → Go to Q3
2. What sources did you use? (multiple answers possible)
   ☐ GLUTENSCREEN website
   ☐ Dutch Celiac Association website
   ☐ Other websites
   ☐ Brochures/books/articles
   ☐ Discussions with others
   ☐ Asked questions to doctors
   ☐ Other: ______

How have you felt over the past week?

3. I feel tense:
   ☐ Usually ☐ Often ☐ Sometimes ☐ Not at all
4. I can sit and relax:
   ☐ Certainly ☐ Mostly ☐ Not often ☐ Not at all
5. I feel worried:
   ☐ Very often ☐ Often ☐ Sometimes ☐ Occasionally
6. I feel cheerful:
   ☐ Not at all ☐ Not often ☐ Sometimes ☐ Usually
7. Do you understand that a positive finger prick result does NOT necessarily mean your child has celiac disease?
   ☐ Yes ☐ No ☐ No opinion
8. Are you satisfied with the information received about the hospital testing?
   ☐ Yes ☐ No ☐ No opinion
9. Would you participate in another early screening in the future?
   ☐ Yes ☐ No ☐ No opinion

At this time, it is still not possible in the Netherlands to test all children (with and without symptoms) who visit the Youth Health Care Center for celiac disease. Currently, testing for celiac disease is only allowed when symptoms are present.

10. Do you think mass screening for celiac disease is a good idea?
    ☐ Yes ☐ No ☐ No opinion
11. Would you have tested your child without symptoms, knowing 40% of cases show none?
    ☐ Yes ☐ No ☐ No opinion

Explain your answer to question 11:
[Open field]

12. Did you take time off work for this appointment?
    ☐ Yes → Go to Q13 ☐ No
13. If yes, how much time? ___ hours ___ minutes

Questions or comments?
[Open field]

Thank you for your cooperation!

Questionnaire 6 – With Complaints, Positive POC Test, Hospital Says NO Celiac Disease

Dear Parent(s)/Guardian(s),

At the Youth Health Care Center, your child recently had a finger prick to measure antibodies against gluten. This was done because your child has one or more symptoms that could be related to celiac disease, which is a gluten intolerance.

The test indicated that your child might have celiac disease. For further investigation, your child was referred to the hospital. Follow-up testing at the hospital did not confirm celiac disease.

These questions are about your opinion on early detection of celiac disease. Completing the questionnaire takes no more than 5 minutes and may be done anonymously.

For questions, contact:

- JGZ Kennemerland: (phone number present in original questionnaire)
- LUMC: (e-mail address and phone number present in original questionnaire)

Thank you very much for your time and cooperation

Demographic Information:

Name of child: ______ Age: (years/months): ______ Sex: ☐ boy ☐ girl
Country of Birth: Parent 1 ______ Parent 2 ______

We would like to know your opinion about the study 'early detection of celiac disease at Youth Health Care Centers,' GLUTENSCREEN. Please check the box that best matches your answer for the following questions. At the end of the questionnaire, there is space for additional questions/comments.

Survey Questions

1. Has your opinion about early celiac screening changed?
   ☐ Yes ☐ No ☐ No opinion
2. After the final result, did you seek more information?
   ☐ Yes → Go to Q3 ☐ No → Go to Q4
3. What sources did you use? (check all that apply)
   ☐ GLUTENSCREEN website
   ☐ Dutch Celiac Association website
   ☐ Other websites
   ☐ Brochures/books/articles
   ☐ Talked with others
   ☐ Consulted doctors
   ☐ Other: ______

Feelings in the past week

4. I feel tense:
   ☐ Usually ☐ Often ☐ Sometimes ☐ Not at all
5. I can sit and relax:
   ☐ Certainly ☐ Mostly ☐ Not often ☐ Not at all
6. I feel worried:
   ☐ Very often ☐ Often ☐ Sometimes ☐ Occasionally
7. I feel cheerful:
   ☐ Not at all ☐ Not often ☐ Sometimes ☐ Usually
8. How did you feel when you heard your child does NOT have celiac disease?
   Not shocked ☐ ☐ ☐ ☐ ☐ Very shocked
   Not worried ☐ ☐ ☐ ☐ ☐ Very worried
   Not anxious ☐ ☐ ☐ ☐ ☐ Very anxious
   Not unhappy ☐ ☐ ☐ ☐ ☐ Very unhappy
   Reassured ☐ ☐ ☐ ☐ ☐ Not reassured
   Relieved ☐ ☐ ☐ ☐ ☐ Not relieved
9. Was it clear that a positive test at the Youth Health Care Center might still mean NO celiac disease (false positive)?
   ☐ Yes ☐ No ☐ No opinion
10. Are you satisfied with the information about the hospital testing?
   ☐ Yes ☐ No ☐ No opinion
11. Would you take part in another screening for celiac disease in the future?
   (Note: A rapid test for celiac antibodies was used to determine whether your child currently has celiac disease. A negative test means that your child does not have celiac disease at this moment, but it does not rule out the possibility that your child could develop it in the future)
   ☐ Yes ☐ No ☐ No opinion

At this time, it is still not possible in the Netherlands to test all children (with and without symptoms) who visit the Youth Health Care Center for celiac disease. Currently, testing for celiac disease is only allowed when symptoms are present.

12. Do you think mass screening for celiac disease is a good idea?
   ☐ Yes ☐ No ☐ No opinion
13. Would you have your child tested without symptoms, knowing 40% of cases are symptom-free?
   ☐ Yes ☐ No ☐ No opinion

Explain your answer to question 13:
[Open field]

Questions or comments?
[Open field]

Thank you for your cooperation!

Questionnaire 7 – With Complaints, Positive POC Test, Hospital Confirms Celiac Disease

Dear Parent(s)/Guardian(s),

At the Youth Health Care Center, your child recently had a finger prick to measure antibodies against gluten. This was done because your child has one or more symptoms that could be associated with celiac disease. Celiac disease is a sensitivity to gluten.

This test indicated that your child might have celiac disease. For further examination, your child was referred to the hospital. Follow-up testing at the hospital confirmed the diagnosis of celiac disease.

These questions are about your opinion on early detection of celiac disease. Completing the questionnaire takes no more than 5 minutes.

For questions, contact:

- JGZ Kennemerland: (phone number present in original questionnaire)
- LUMC: (e-mail address and phone number present in original questionnaire)

Thank you very much for your time and cooperation

Demographic Information:

Name of child: ______ Age: (years/months): ______ Sex: ☐ boy ☐ girl
Country of Birth: Parent 1 ______ Parent 2 ______

We would like to know your opinion about the study 'early detection of celiac disease at Youth Health Care Centers,' GLUTENSCREEN. Please check the box that best matches your answer for the following questions. At the end of the questionnaire, there is space for additional questions/comments.

Survey Questions

1. Has your opinion about early screening changed?
   ☐ Yes ☐ No ☐ No opinion
2. After the confirmed diagnosis, did you seek additional information?
   ☐ Yes → Go to Q3
   ☐ No → Go to Q4
3. What sources did you use? (check all that apply)
   ☐ GLUTENSCREEN website
   ☐ Dutch Celiac Association website
   ☐ Other websites
   ☐ Brochures/books/articles
   ☐ Talked with others
   ☐ Consulted doctors
   ☐ Other: ______

Feelings in the past week

4. I feel tense:
   ☐ Usually ☐ Often ☐ Sometimes ☐ Not at all
5. I can sit and relax:
   ☐ Certainly ☐ Mostly ☐ Not often ☐ Not at all
6. I feel worried:
   ☐ Very often ☐ Often ☐ Sometimes ☐ Occasionally
7. I feel cheerful:
   ☐ Not at all ☐ Not often ☐ Sometimes ☐ Usually
8. How did you feel after the diagnosis?
   Not shocked ☐ ☐ ☐ ☐ ☐ Very shocked
   Not worried ☐ ☐ ☐ ☐ ☐ Very worried
   Not anxious ☐ ☐ ☐ ☐ ☐ Very anxious
   Not unhappy ☐ ☐ ☐ ☐ ☐ Very unhappy
   Reassured ☐ ☐ ☐ ☐ ☐ Not reassured
   Relieved ☐ ☐ ☐ ☐ ☐ Not relieved
9. Are you satisfied with the information from LUMC?
   ☐ Yes ☐ No ☐ No opinion

At this time, it is still not possible in the Netherlands to test all children (with and without symptoms) who visit the Youth Health Care Center for celiac disease. Currently, testing for celiac disease is only allowed when symptoms are present.

10. Do you think mass screening for celiac disease is a good idea?
    ☐ Yes ☐ No ☐ No opinion
11. Would you have tested your child WITHOUT symptoms, knowing 40% of cases are symptom-free? ☐ Yes ☐ No ☐ No opinion

Explain your answer to question 11:
[Open field]

Questions or comments?
[Open field]

Thank you for your cooperation!

Questionnaire for health care professionals

Part 1

It is clear to me what the importance of GLUTENSCREEN is	□ Completely disagree<br>□ Disagree<br>□ Neutral<br>□ Agree<br>□ Completely agree<br>□ Not applicable
It is clear to me which activities and in which order I have to do for GLUTENSCREEN	□ Completely disagree<br>□ Disagree<br>□ Neutral<br>□ Agree<br>□ Completely agree<br>□ Not applicable
Training is necessary for my task within GLUTENSCREEN	□ Completely disagree<br>□ Disagree<br>□ Neutral<br>□ Agree<br>□ Completely agree<br>□ Not applicable
My JGZ organization gives me enough time to carry out the activities for GUTENSCREEN in my daily work	□ Completely disagree<br>□ Disagree<br>□ Neutral<br>□ Agree<br>□ Completely agree<br>□ Not applicable
The location where I work is sufficiently equipped to properly carry out GLUTENSCREEN	□ Completely disagree<br>□ Disagree<br>□ Neutral<br>□ Agree<br>□ Completely agree<br>□ Not applicable
The CD-associated symptom questionnaire used within GLUTENSCREEN is user-friendly	□ Completely disagree<br>□ Disagree<br>□ Neutral<br>□ Agree<br>□ Completely agree<br>□ Not applicable
I can better help parents who are concerned about complaints described in the GLUTENSCREEN questionnaire by offering a POC test (finger prick)	□ Completely disagree<br>□ Disagree<br>□ Neutral<br>□ Agree<br>□ Completely agree<br>□ Not applicable
The CD-associated symptom questionnaire can be incorporated into the regular consultation	□ Completely disagree<br>□ Disagree<br>□ Neutral<br>□ Agree

	□ Completely agree<br>□ Not applicable
It takes me little effort to find parents willing to participate in the CD-associated symptom questionnaire	□ Completely disagree<br>□ Disagree<br>□ Neutral<br>□ Agree<br>□ Completely agree<br>□ Not applicable
The questionnaires about the acceptance of case finding for CD are user-friendly	□ Completely disagree<br>□ Disagree<br>□ Neutral<br>□ Agree<br>□ Completely agree<br>□ Not applicable
It takes me little effort to find parents willing to participate in the acceptance questionnaires	□ Completely disagree<br>□ Disagree<br>□ Neutral<br>□ Agree<br>□ Completely agree<br>□ Not applicable
The POC test (finger prick) is user-friendly	□ Completely disagree<br>□ Disagree<br>□ Neutral<br>□ Agree<br>□ Completely agree<br>□ Not applicable
Sufficient time is available to perform the POC test	□ Completely disagree<br>□ Disagree<br>□ Neutral<br>□ Agree<br>□ Completely agree<br>□ Not applicable
I am good at communicating the results of the POC test to parents	□ Completely disagree<br>□ Disagree<br>□ Neutral<br>□ Agree<br>□ Completely agree<br>□ Not applicable
In case of a positive POC result, a youth doctor can be consulted the same day	□ Completely disagree<br>□ Disagree<br>□ Neutral<br>□ Agree<br>□ Completely agree<br>□ Not applicable
It takes me little effort to find parents willing to participate in the POC test	□ Completely disagree<br>□ Disagree<br>□ Neutral<br>□ Agree<br>□ Completely agree<br>□ Not applicable

In carrying out GLUTENSCREEN, I receive sufficient support from my direct colleagues	□ Completely disagree<br>□ Disagree<br>□ Neutral<br>□ Agree<br>□ Completely agree<br>□ Not applicable
I have the impression that GLUTENSCREEN is supported by Youth Health Care Kennemerland	□ Completely disagree<br>□ Disagree<br>□ Neutral<br>□ Agree<br>□ Completely agree<br>□ Not applicable
The progress/outcome of GLUTENSCREEN is being communicated to me	□ Completely disagree<br>□ Disagree<br>□ Neutral<br>□ Agree<br>□ Completely agree<br>□ Not applicable

Part 2

Early detection of celiac disease adds value to the care we provide as Youth Health Care Centre to children aged 1-4 years	
I can answer questions from parents about early detection of celiac disease well	
If I have questions about (early detection of) celiac disease, I know where to find information	
In the future, the POC test can be performed during a regular consultation with the youth doctor/nurse (NB: two signatures are not required for regular care).	
The POC test can take place in a separate consultation in the future	
I have the impression that the early detection of celiac disease is supported by the professional associations and employers within the Youth Health Care	
