# Supplementary material for: Acceptability of screening for celiac disease at Youth Health Care Centers in The Netherlands
Source: Eur J Pediatr. 2026 Apr 6;185(5):237. doi: 10.1007/s00431-026-06809-6 (PMC13053595; doi:10.1007/s00431-026-06809-6)
Supplement: Supplementary file 2 — (DOCX 25.0 KB) [file 431_2026_6809_MOESM2_ESM.docx]

**Appendix 2**

1. Does your child have abdominal pain for more than 3 weeks (at least twice a week)?
2. Does your child have abdominal bloating?
3. Does your child regularly have constipation not responsive to laxatives?
4. Does your child have diarrhea for more than 14 days?
5. Has your child been spitting up for more than 3 weeks (at least twice a week)?
6. Do you find your child easily tired to the point that he / she is hindered in daily activities?
7. Does your child regularly have aphthous stomatitis (mouth ulcers)?
8. Is your child regularly irritated (longer than 3 weeks, at least 2x weeks)?
9. Is growth (height and / or weight) restricted?
10. Does your child eat gluten?
11. Has your child been diagnosed with coeliac disease?

| **Appendix 3** – Questionnaire responses per questionnaire item | | | | | | | |
| --- | --- | --- | --- | --- | --- | --- | --- |
|  | **Questionnaire 1** | **Questionnaire 2*** | **Questionnaire 3*** | **Questionnaire 4** | **Questionnaire 5** | **Questionnaire 6** | **Questionnaire 7** |
| **Questionnaires total** | **1070** | **653** | **259** | **1095** | **39** | **2** | **32** |
| **Age child** | 802 | 411 | 151 | 970 | 34 | 1 | 26 |
| **Gender child** | 969 | 443 | 168 | 1070 | 38 | 1 | 30 |
| **Educational level parent 1** | 1060 | 449 | 173 | 1083 | 17 | 0 | 14 |
| **Educational level parent 2** | 1023 | 431 | 165 | 1045 | 17 | 0 | 14 |
| **Country of birth parent 1** | 783 | 432 | 152 | 1032 | 36 | 1 | 30 |
| **Country of birth parent 2** | 774 | 421 | 143 | 996 | 36 | 1 | 30 |
| **Worried about child’s health** | 1061 | 640 | 258 | 1083 | - | - | - |
| **Parental suspicion of CD** | 1063 | 647 | 259 | 1078 | - | - | - |
| **Would let your child participate if symptomatic** | 1055 | - | - | - | - | - | - |
| **Mass screening is a good idea** | 1060 | 647 | 253 | 1079 | 39 | 2 | 32 |
| **Test children without symptoms** | 1057 | 646 | 250 | 1085 | 39 | 2 | 32 |
| **Open question about mass screening** | 1062 | 652 | 258 | 1092 | 39 | 2 | 32 |
| **Received sufficient information about study** | - | 640 | 251 | 1032 | - | - | - |
| **Reason for declining participation** | - | - | 240 | - | - | - | - |
| **Participate if POC test was immediately** | - | - | 248 | - | - | - | - |
| **Confidence in test** | - | - | - | 1088 | - | - | - |
| **Worried about result** | - | - | - | 1086 | - | - | - |
| **Rating information** | - | - | - | 1067 | - | - | - |
| **Result POC test** | - | - | - | 1082 | - | - | - |
| **Feeling worried after POC test result** | - | - | - | 758 | - | 2 | - |
| **Feeling concerned after POC test result** | - | - | - | 742 | - | 2 | - |
| **Feeling anxious after POC test result** | - | - | - | 733 | - | 2 | - |
| **Feeling unhappy after POC test result** | - | - | - | 729 | - | 2 | - |
| **Feeling (not)Reassured after POC test result** | - | - | - | 886 | - | 2 | - |
| **Feeling (not)Relieved after POC test result** | - | - | - | 863 | - | 2 | - |
| **Participation in future** | - | - | - | 1083 | 39 | 2 | - |
| **HADS – I felt tense** | - | - | - | - | 39 | 2 | 32 |
| **HADS – I felt relaxed** | - | - | - | - | 39 | 2 | 32 |
| **HADS – I felt worried** | - | - | - | - | 39 | 2 | 32 |
| **HADS – I felt cheerful** | - | - | - | - | 39 | 2 | 32 |
| * For questionnaires 2 and 3, of N=202 and N=71 participants respectively, demographic information (age and gender of the child, educational level and country of birth of the parents) was missing due to informed consent for anonymized data processing. | | | | | | | |

| ***Appendix 4 –*** *Answers of parents about mass screening for asymptomatic children in the various studied groups* | | | | | | | | | | | | | | |
| --- | --- | --- | --- | --- | --- | --- | --- | --- | --- | --- | --- | --- | --- | --- |
|  | Questionnaire 1 | | Questionnaire 2 | | Questionnaire 3 | | Questionnaire 4 | | Questionnaire 5 | | Questionnaire 6 | | Questionnaire 7 | |
|  | Would not test (n=125) | Would test (n=700) | Would not test (n= 80) | Would test (n=461) | Would not test (n=67) | Would test (n=84) | Would not test (n=97) | Would test (n=819) | Would not test (n= 2 ) | Would test (n=33) | Would not test (n=0) | Would test (n=1) | Would not test (n= 3) | Would test (n=25) |
| Individual certainty* | 22 (28/125) | 31 (220/700) | 13 (10/80) | 23 (105/461) | 19 (13/67) | 30 (25/84) | 18 (17/97) | 36 (297/819) | 0 | 30 (10/33) |  | 0 | 33 (1/3) | 28 (7/25) |
| Concerning diagnostics* | (17/125) 14 | 17 (122/700) | 30 (24/80) | 18 (82/461) | 19 (13/67) | 17 (14/84) | 29 (28/97) | 15 (123/819) | 50 (1/2) | 6 (2/33) |  | 0 | 33 (1/3) | 44 (11/25) |
| Consequence for future* | 0 (0/125) | 7 (47/700) | 1 (1/80) | 15 (70/461) | 0 (0/67) | 4 (3/84) | 0 (0/97) | 9 (77/819) | 0 | 9 (3/33) |  | 0 | 0 | 12 (3/25) |
| For research and science* | 6 (7/125) | 6 (43/700) | 0 (0/80) | 4 (17/461) | 0 (0/67) | 5 (4/84) | 1 (1/97) | 2 (16/819) | 0 | 0 (0/33) |  | 0 | 0 | 0 (0/25) |
| For other children* | 0 (0/125) | 2 (16/700) | 0 (0/80) | 1 (4/461) | 0 (0/67) | 1 (1/84) | 1 (1/97) | 2 (16/819) | 0 | 3 (1/33) |  | 100 (1/1) | 0 | 0 (0/25) |
| Other* | 22 (28/125) | 15 (107/700) | 33 (26/80) | 17 (77/461) | 25 (17/64) | 21 (18/84) | 13 (13/97) | 10 (83/819) | 0 | 6 (2/33) |  | 0 | 33 (1/3) | 12 (3/25) |
| No opinion / no answer given | 36 (45/125) | 21 (145/700) | 24 (29/80) | 23 (106/461) | 36 (24/67) | 23 (19/84) | 38 (38/97) | 25 (207/819) | 50 (1/2) | 45 (15/33) |  | 0 | 0 | 4 (1/25) |
| ** Explanation of categories: individual certainty – all answers that indicated a motive for their own child; concerning diagnostics – all answers that included an opinion on the POC test or having symptoms; consequence for future – all answers that included a beneficial or disadvantageous consequence for the future; for research and science – all answers that indicated the importance of research and science; for other children – all answers that indicated the importance of testing for other children, other – individual arguments no to be categorized in the aforementioned categories.* | | | | | | | | | | | | | | |
