# Supplementary material for: Acceptability of screening for celiac disease at Youth Health Care Centers in The Netherlands
Source: Eur J Pediatr. 2026 Apr 6;185(5):237. doi: 10.1007/s00431-026-06809-6 (PMC13053595; doi:10.1007/s00431-026-06809-6)
Supplement: Supplementary file 3 — (DOCX 16.7 KB) [file 431_2026_6809_MOESM3_ESM.docx]

**Appendix 3**

| **Appendix 3** – Questionnaire responses per questionnaire item | | | | | | | |
| --- | --- | --- | --- | --- | --- | --- | --- |
|  | **Questionnaire 1** | **Questionnaire 2*** | **Questionnaire 3*** | **Questionnaire 4** | **Questionnaire 5** | **Questionnaire 6** | **Questionnaire 7** |
| **Questionnaires total** | **1070** | **653** | **259** | **1095** | **39** | **2** | **32** |
| **Age child** | 802 | 411 | 151 | 970 | 34 | 1 | 26 |
| **Gender child** | 969 | 443 | 168 | 1070 | 38 | 1 | 30 |
| **Educational level parent 1** | 1060 | 449 | 173 | 1083 | 17 | 0 | 14 |
| **Educational level parent 2** | 1023 | 431 | 165 | 1045 | 17 | 0 | 14 |
| **Country of birth parent 1** | 783 | 432 | 152 | 1032 | 36 | 1 | 30 |
| **Country of birth parent 2** | 774 | 421 | 143 | 996 | 36 | 1 | 30 |
| **Worried about child’s health** | 1061 | 640 | 258 | 1083 | - | - | - |
| **Parental suspicion of CD** | 1063 | 647 | 259 | 1078 | - | - | - |
| **Would let your child participate if symptomatic** | 1055 | - | - | - | - | - | - |
| **Mass screening is a good idea** | 1060 | 647 | 253 | 1079 | 39 | 2 | 32 |
| **Test children without symptoms** | 1057 | 646 | 250 | 1085 | 39 | 2 | 32 |
| **Open question about mass screening** | 1062 | 652 | 258 | 1092 | 39 | 2 | 32 |
| **Received sufficient information about study** | - | 640 | 251 | 1032 | - | - | - |
| **Reason for declining participation** | - | - | 240 | - | - | - | - |
| **Participate if POC test was immediately** | - | - | 248 | - | - | - | - |
| **Confidence in test** | - | - | - | 1088 | - | - | - |
| **Worried about result** | - | - | - | 1086 | - | - | - |
| **Rating information** | - | - | - | 1067 | - | - | - |
| **Result POC test** | - | - | - | 1082 | - | - | - |
| **Feeling worried after POC test result** | - | - | - | 758 | - | 2 | - |
| **Feeling concerned after POC test result** | - | - | - | 742 | - | 2 | - |
| **Feeling anxious after POC test result** | - | - | - | 733 | - | 2 | - |
| **Feeling unhappy after POC test result** | - | - | - | 729 | - | 2 | - |
| **Feeling (not)Reassured after POC test result** | - | - | - | 886 | - | 2 | - |
| **Feeling (not)Relieved after POC test result** | - | - | - | 863 | - | 2 | - |
| **Participation in future** | - | - | - | 1083 | 39 | 2 | - |
| **HADS – I felt tense** | - | - | - | - | 39 | 2 | 32 |
| **HADS – I felt relaxed** | - | - | - | - | 39 | 2 | 32 |
| **HADS – I felt worried** | - | - | - | - | 39 | 2 | 32 |
| **HADS – I felt cheerful** | - | - | - | - | 39 | 2 | 32 |
| * For questionnaires 2 and 3, of N=202 and N=71 participants respectively, demographic information (age and gender of the child, educational level and country of birth of the parents) was missing due to informed consent for anonymized data processing. | | | | | | | |
