# Supplementary material for: Acceptability of screening for celiac disease at Youth Health Care Centers in The Netherlands
Source: Eur J Pediatr. 2026 Apr 6;185(5):237. doi: 10.1007/s00431-026-06809-6 (PMC13053595; doi:10.1007/s00431-026-06809-6)
Supplement: Supplementary file 4 — (DOCX 16.5 KB) [file 431_2026_6809_MOESM4_ESM.docx]

**Appendix 4**

| ***Appendix 4 –*** *Answers of parents about mass screening for asymptomatic children in the various studied groups* | | | | | | | | | | | | | | |
| --- | --- | --- | --- | --- | --- | --- | --- | --- | --- | --- | --- | --- | --- | --- |
|  | Questionnaire 1 | | Questionnaire 2 | | Questionnaire 3 | | Questionnaire 4 | | Questionnaire 5 | | Questionnaire 6 | | Questionnaire 7 | |
|  | Would not test (n=125) | Would test (n=700) | Would not test (n= 80) | Would test (n=461) | Would not test (n=67) | Would test (n=84) | Would not test (n=97) | Would test (n=819) | Would not test (n= 2 ) | Would test (n=33) | Would not test (n=0) | Would test (n=1) | Would not test (n= 3) | Would test (n=25) |
| Individual certainty* | 22 (28/125) | 31 (220/700) | 13 (10/80) | 23 (105/461) | 19 (13/67) | 30 (25/84) | 18 (17/97) | 36 (297/819) | 0 | 30 (10/33) |  | 0 | 33 (1/3) | 28 (7/25) |
| Concerning diagnostics* | (17/125) 14 | 17 (122/700) | 30 (24/80) | 18 (82/461) | 19 (13/67) | 17 (14/84) | 29 (28/97) | 15 (123/819) | 50 (1/2) | 6 (2/33) |  | 0 | 33 (1/3) | 44 (11/25) |
| Consequence for future* | 0 (0/125) | 7 (47/700) | 1 (1/80) | 15 (70/461) | 0 (0/67) | 4 (3/84) | 0 (0/97) | 9 (77/819) | 0 | 9 (3/33) |  | 0 | 0 | 12 (3/25) |
| For research and science* | 6 (7/125) | 6 (43/700) | 0 (0/80) | 4 (17/461) | 0 (0/67) | 5 (4/84) | 1 (1/97) | 2 (16/819) | 0 | 0 (0/33) |  | 0 | 0 | 0 (0/25) |
| For other children* | 0 (0/125) | 2 (16/700) | 0 (0/80) | 1 (4/461) | 0 (0/67) | 1 (1/84) | 1 (1/97) | 2 (16/819) | 0 | 3 (1/33) |  | 100 (1/1) | 0 | 0 (0/25) |
| Other* | 22 (28/125) | 15 (107/700) | 33 (26/80) | 17 (77/461) | 25 (17/64) | 21 (18/84) | 13 (13/97) | 10 (83/819) | 0 | 6 (2/33) |  | 0 | 33 (1/3) | 12 (3/25) |
| No opinion / no answer given | 36 (45/125) | 21 (145/700) | 24 (29/80) | 23 (106/461) | 36 (24/67) | 23 (19/84) | 38 (38/97) | 25 (207/819) | 50 (1/2) | 45 (15/33) |  | 0 | 0 | 4 (1/25) |
| ** Explanation of categories: individual certainty – all answers that indicated a motive for their own child; concerning diagnostics – all answers that included an opinion on the POC test or having symptoms; consequence for future – all answers that included a beneficial or disadvantageous consequence for the future; for research and science – all answers that indicated the importance of research and science; for other children – all answers that indicated the importance of testing for other children, other – individual arguments no to be categorized in the aforementioned categories.* | | | | | | | | | | | | | | |
